# Leaky Sewers Hydraulically Disconnect from Groundwater: A Proof‐of‐Concept

**DOI:** 10.1111/gwat.70083

**Published:** 2026-06-02

**Authors:** Aaron Peche, Fritz Kalwa, Syed Mohaiminul Islam, Georg Houben, Thomas Graf, Sven Altfelder

**Affiliations:** ^1^ Federal Institute for Geosciences and Natural Resources (BGR) Stilleweg 2 D‐30655 Hannover Germany; ^2^ Leibniz University Hannover Institute of Fluid Mechanics and Environmental Physics in Civil Engineering Hannover Germany

## Abstract

Leakage from aging sewer and stormwater pipes into the subsurface poses significant environmental risks and threatens the integrity of urban infrastructure, primarily through the degradation of groundwater quality and the alteration of urban water balances. While the presence of defects within pipe networks is well‐documented, accurately quantifying volumetric exchange fluxes remains a challenge due to the complex, nonlinear interactions between the pipe, the surrounding variably saturated soil, and the fluctuating groundwater level. Current modeling approaches often overlook the threshold behaviors of these systems, leading to potential inaccuracies in leakage estimation. In this study, we show for the first time that leaky pipes can become hydraulically disconnected from the underlying groundwater, a phenomenon analogous to well‐known river–groundwater interactions that include disconnection. In a sewer–groundwater context, hydraulic disconnection is restricted to point sources and is strongly influenced by colmation/clogging. Through numerical modeling of a hypothetical case study, we show that the leakage flux from the pipe (in absolute terms) initially increases with declining groundwater levels, until a critical depth below the leaky pipe is reached. After this point, the hydraulic communication from the groundwater to the leaky pipe stops and the leakage flux can be considered constant. In a sensitivity analysis, we demonstrate the impact of the individual hydraulic parameters of the leaky‐pipe‐groundwater system on the hydraulic disconnection. We further modify the properties of the aquifer material, resulting in a hydraulic disconnection depth of 0.89 m, 1.77 m and 4.00 m below pipe for sand, loamy sand and sandy loam aquifers, respectively. This insight has important implications for leakage modeling: once hydraulic disconnection occurs, the leakage flux becomes independent of groundwater dynamics. The present study provides a proof‐of‐concept for the mechanism by which leaky sewers hydraulically disconnect from groundwater.

## Graphical abstract

In this study, we newly demonstrate that leaky pipes can become hydraulically disconnected from the underlying groundwater—a phenomenon analogous to river–groundwater interactions. Through numerical modeling, we show that, as the groundwater table declines, the leakage flux from the pipe (in absolute terms) initially increases until a critical depth between the leaky pipe and the groundwater table is reached. Beyond this depth, the flux stabilizes and becomes constant.

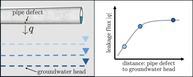

## Introduction

Subsurface wastewater pipes do not only perform their intended function of disposing of sewage, but—due to defects and together with their trenches and surrounding bedding material—also interact with the surrounding soils and aquifers in many ways. Due to their abundance, especially in urban environments, such systems are often referred to as “urban karst” (Bonneau et al. 2017). Natural material aging in combination with pipe corrosion through a complex interaction of chemical, environmental, and biological factors (Mori et al. 1992; Li et al. 2023), differential ground movement or excessive loads (Damvergis 2014) may lead to defect clusters or cracks in the pipe network components. When subsurface pipes are defective, exchange fluxes between pipe water and groundwater/the surrounding soil occur in both directions. In the first case and when the groundwater level is above the pipe water level, groundwater infiltrates into defect pipes. This process may lead to reduced pipe system capacity and treatment efficiency (Ellis 2001; Karpf and Krebs 2013) and significant alterations of the urban water balance. Pipe infiltration may take up the bulk of groundwater recharge, and groundwater levels may be lower close to the level of the pipe network (Peche et al. 2019). In the other case of exfiltration from pipes, which occurs when the pipe water level is above the groundwater level, untreated stormwater and sewage may reach the groundwater zone. This poses a hydrochemical and microbiological threat, especially in areas where groundwater is a potable resource (DeSilva et al. 2005). Characterizing the physical mechanisms of sewer leakage is critical for developing effective mitigation strategies. This study investigates these processes, focusing specifically on the sewer exfiltration.

Sewer leakage varies in its form and extent. It often depends on the state of maintenance of the pipe network. In terms of relative values, sewer leakage may add up to 1–13% of total pipe flow, but due to the potential impact on groundwater quality, this process is considered highly relevant (Rutsch et al. 2006; Vroblesky et al. 2011). Unfortunately, studies with actual field data are scarce and difficult to conduct, so there is a need for simulation tools applicable within low data availability.

Pipes with high‐quality water—for example, for drinking or irrigation– usually show an unhindered exfiltration into the surrounding subsurface, while turbid low‐quality waters—for example, from sewers—tend to cause clogging. Clogging can be caused by particles, accumulating at the point of leakage, commonly referred to as “physical clogging,” which is associated to the turbidity of the infiltrating water (Skolasińska 2006; Kalwa et al. 2021). Furthermore, nutrient‐rich waters tend to facilitate microbial growth. This leads to the blocking of flow‐active pores by biofilms, consisting of the microorganisms and their associated extra‐polymeric substances (EPS), which is commonly referred to as “biological clogging” or “bioclogging” (Thullner et al. 2004; Skolasińska 2006; Mostafa and Van Geel 2007). The dynamics of both processes depends highly on hydraulic gradient, flow rate, nutrient/particle load and available pore space (Kalwa et al. 2021).

In the context of sewer exfiltration, it is commonly assumed that, during the initial phase—minutes to hours after exfiltration started—physical clogging at the interface between pipe and soil is the dominant process (Karpf et al. 2009). At a later stage—after days to weeks—biological clogging becomes more dominant and leads to a consolidation and propagation of clogging 10–50 mm into the sediment (Ellis et al. 2009). The effect of the so‐called “cream layer”—formed inside the pipe by shredded toilet paper, sediment and grease—is neglected in this study, due to the unpredictability of its hydraulic properties. Large pipe water outbursts may break the clogging layer and lead to short‐term large leakage, called leakage pulse (Ellis et al. 2009) before the clogging layer forms anew. With regards to the hydraulic properties, the clogging layer is usually characterized by significantly lower values of hydraulic conductivity than the surrounding soil (Rauch and Stegner 1994; Nguyen et al. 2021). In hydraulic calculations, the hydraulic properties of these clogging layers are assumed to be constant (Nguyen et al. 2021).

Pipes are usually embedded in a backfill material, with well‐known properties (Karpf 2012; Nguyen et al. 2021). In Germany, the backfill material is standardized and uniform sand is a typical backfill material (Karpf 2012). The backfill material is surrounded by the natural porous medium (Rutsch et al. 2008). In a vertical one‐dimensional pipe leakage problem with the pipe defect at its topmost, three porous media have to be considered, (1) the clogging or colmation layer, (2) the backfill, and (3) the natural porous medium.

Since physical measurements of flow around leaky sewers and their surrounding porous media are notoriously difficult to obtain, numerical models are essential for quantifying the problem and advancing process understanding. In recent times, different modeling approaches have been used to investigate fundamentals of the sewer leakage process. A summary of some major techniques and approaches is given in Peche (2019), Nguyen et al. (2021), and extended by the methods used in D'Aniello et al. (2026). Generally, empirical approaches and models based on Darcy's linear theory are often restricted and fail to adequately calculate sewer leakage (Rutsch et al. 2008), which highlights the complexity of the problem and the nonlinearity of the physical process. Hence, physically based models for pipe flow and variably saturated flow are required to estimate pipe leakage with sufficient precision (Mohrlok et al. 2008). In a recent study, D'Aniello et al. (2026) compared three different physically based subsurface flow and transport modeling approaches coupled to a semi‐distributed hydrological model for surface and sewer flow. The authors conclude that the adequate representation of the variably saturated zone and the pipe geometry is crucial for the accurate calculation of sewer leakage and associated contaminant transport. In a previous study, D'Aniello et al. (2022) investigated how the utility trenches around a leaking pipe can impact the spreading of leakage water within the subsurface. By employing a numerical simulator, the authors found that, for example, if retention properties of the trench backfill material result in much lower effective water saturation and relative conductivity (due to larger pore diameters and lower suction) than in the surrounding natural soil, trenches rather act as capillary barriers diverting the downward flow within the native soil around the trenches. If, on the other hand, the water saturation in the trenches is large, they act as sinks. This contradicts the general assumption that backfill trenches act as zones for preferential flow (Bonneau et al. 2017). In a study investigating the hydraulic performance of stormwater infiltration ponds, D'Aniello et al. (2019) investigated the impact of lowering the groundwater table on exchange fluxes from the infiltration pond into the subsurface, among others. They find, for all their scenarios, that average exchange fluxes increase nonlinearly with decreasing groundwater level. A similar increase in the exchange flux, however, based on less data, has also been documented by, for example, Duchene et al. (1994). This indicates the typical behavior of a hydraulically connected system developing toward hydraulic disconnection. However, their study did not focus on the hydraulic disconnection. The only study where the hydraulic disconnection of leaky sewers from groundwater is mentioned is a study by Peche et al. (2017). The authors developed a physically based model by coupling a pipe flow simulator with a variably saturated subsurface flow simulator. Among others, the authors analyze the relationship between (1) the leakage flux, (2) the water pressure at the lower boundary of the colmation layer, and (3) the pipe water level, they found that (1) converges toward a constant value at (2) below a certain threshold. The authors argue that this behavior may be due to a hydraulically disconnected system. However, the authors had a different objective and merely assumed that phenomenon. Consequently, and to the best of our knowledge, a proof‐of‐concept of the hydraulic disconnection of leaky sewers from groundwater is missing.

In the river–groundwater context, the process of hydraulic disconnection is widely described in literature (Sophocleous 2002; Brunner et al. 2009; Brunner et al. 2011). It occurs when the groundwater level is sufficiently deep, such that changes in the groundwater level do not alter the infiltration rate from the river (Brunner et al. 2009). Similar to this hydraulic disconnection of river–groundwater systems, leaky pipes can also hydraulically disconnect from groundwater. However, certain differences exist between the river–groundwater and the sewer–groundwater systems. For example, rivers may exhibit large climate‐driven hydraulic variations (e.g., large hydrostatic pressures during a wet period) which may significantly change the hydraulic properties of the riverbed (Rosenberry et al. 2021) and even lead to geomorphological changes (e.g., Barrocu and Eslamian 2022). In contrast to this, sewers are stable infrastructural features and large hydrostatic pressures are capped by unloading excess water through, for example, overflow chambers or spillways. Also, sewers are usually located below impervious urban surfaces, affecting specific groundwater dynamics which may differ greatly from groundwater dynamics under a river.

The process of hydraulic disconnection occurs due to a complex interplay between soil water saturation—affecting the relative permeability—and the hydraulic gradient. A detailed definition of this problem with regards to the interplay of the saturation, relative permeability and hydraulic gradient, is provided below.

**Figure A1:**
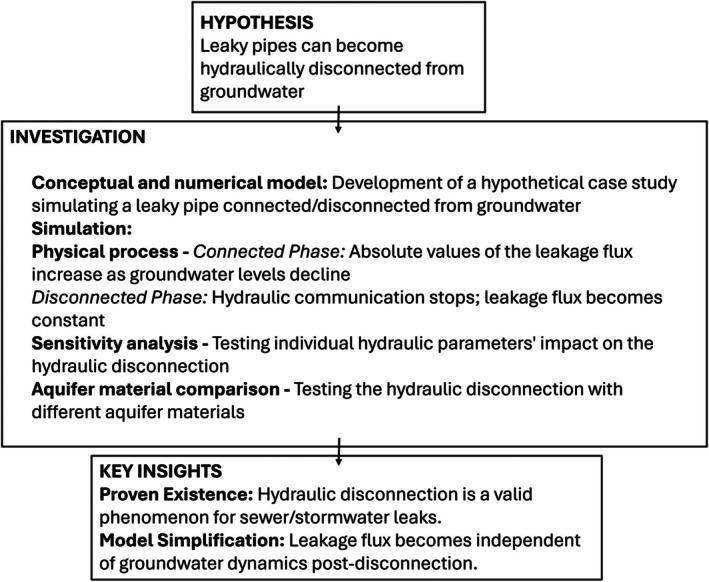
Methodological flowchart including key insights of the present study.

In this work, we provide a proof‐of‐concept that leaky sewers hydraulically disconnect from groundwater under certain circumstances. For that purpose, we employ a numerical simulator to show that leakage rates converge to a constant value with decreasing groundwater levels. We further show sensitivity of the hydraulic parameters and thicknesses of the individual porous media and the pipe water level. The general concept is applicable to further subsurface pipe infrastructures, such as stormwater pipes and drinking water mains. A methodological flowchart of this work is given as Figure A1 in the Appendix.

## Methods

### Qualitative Assessment of Hydraulic Disconnection—The Interplay of Saturation, Relative Permeability, and Hydraulic Gradient

In order to qualitatively analyze the process of hydraulic disconnection, let us consider a single‐layer problem where the unsaturated water flux in a variably saturated medium is given by:

(1) $$q=K_{r}K\cdot |\nabla H|$$

where q (L/T) is unsaturated water flux from the leaky pipe to the porous medium, that is, the leakage flux, $K_{r}=f\left(S\right)$ (‐) is the relative permeability as a function of effective saturation S (‐), K (L/T) is the saturated hydraulic conductivity and ∣∇H∣ (‐) is the hydraulic gradient (sum of matric potential gradient and geodetic gradient) as an absolute value. When monitoring the value of ∣∇H∣ at a constant reference depth, while the groundwater level is lowered simultaneously, we observe that ∣∇H∣ increases while at the same time $K_{r}K$ decreases, due to the drainage of pores and decreasing saturation S. In a single porous media layer‐problem, this dynamic is limited by ∣∇H∣ reaching unit gradient, resulting in free drainage where $K_{r}K$ is equal to the flux. While groundwater levels (and hence ∣∇H∣) usually vary in one or possibly two orders of magnitude, $K_{r}K$ can vary by many more orders of magnitude, as the function is nonlinear and usually spans over several orders of magnitude. For example, Peche et al. (2017) demonstrate this for a three‐dimensional, three‐layered pipe leakage problem with colmation layer and backfill material above a sandy natural porous medium. In that study, lowering the groundwater level from 0.075 to 0.3 m below the leaky pipe leads to an increase of ∣∇H∣ within the same order of magnitude and a decrease of $K_{r}K$ by four orders of magnitude.

Clearly, this interplay is nonlinear due to the nonlinear nature of the physical process of variably saturated flow and in a multi‐layer problem it also strongly depends on the hydraulic properties of the different porous media. With a Dirichlet boundary condition at the top of the colmation layer, generally, K of the colmation layer majorly controls the saturated flux in its upper part. While approaching the various interfaces between the different layers, saturation systematically decreases or increases and so does ∣∇H∣. These changes ensure that flux remains constant over depth at the same time accounting for the different material properties of the underlying materials. Upon lowering the groundwater level in a system with a still hydraulically connected pipe S in the lowest and thickest layer—the natural porous medium—will asymptotically approach a minimum value for saturation and at the same time ∣∇H∣ approaches unit gradient when monitored at a constant reference depth. Eventually q will reach a maximum value equal to $K_{r}K$ and stay constant with further decrease of the groundwater level leading to hydraulic disconnection. In the present study, we will refer to the minimum vertical distance between the pipe defect and the groundwater level, at which a constant q first develops (and thereby hydraulic disconnection) as “hydraulic disconnection depth.”

### Numerical Simulator and Mathematical Model

The multiphysics simulator OpenGeoSys (version 5.5) (Kolditz et al. 2012) is used for all simulations. This freeware open‐source numerical simulator employs the centered Galerkin Finite Element Method (FEM) to discretize the Richards (1931) equation for unsaturated flow in porous media. The van Genuchten (1980) parameterization is used for the relative conductivity function and retention function. The potential‐based form of the Richards equation is used. For different forms, such as the water‐content‐based or the mixed form, see for example, Zha et al. (2019). A detailed description of the mathematical model is given in the Appendix and in Kolditz et al. (2016). The simulator is well tested on numerous benchmarks (e.g., Kolditz et al. 2016), also for benchmarks of the pipe leakage problem in variably saturated soil (Peche et al. 2017; Peche and Graf 2021).

### Conceptual Model, Material Properties of the Base Case and Sensitivity Analysis

**Figure 1:**
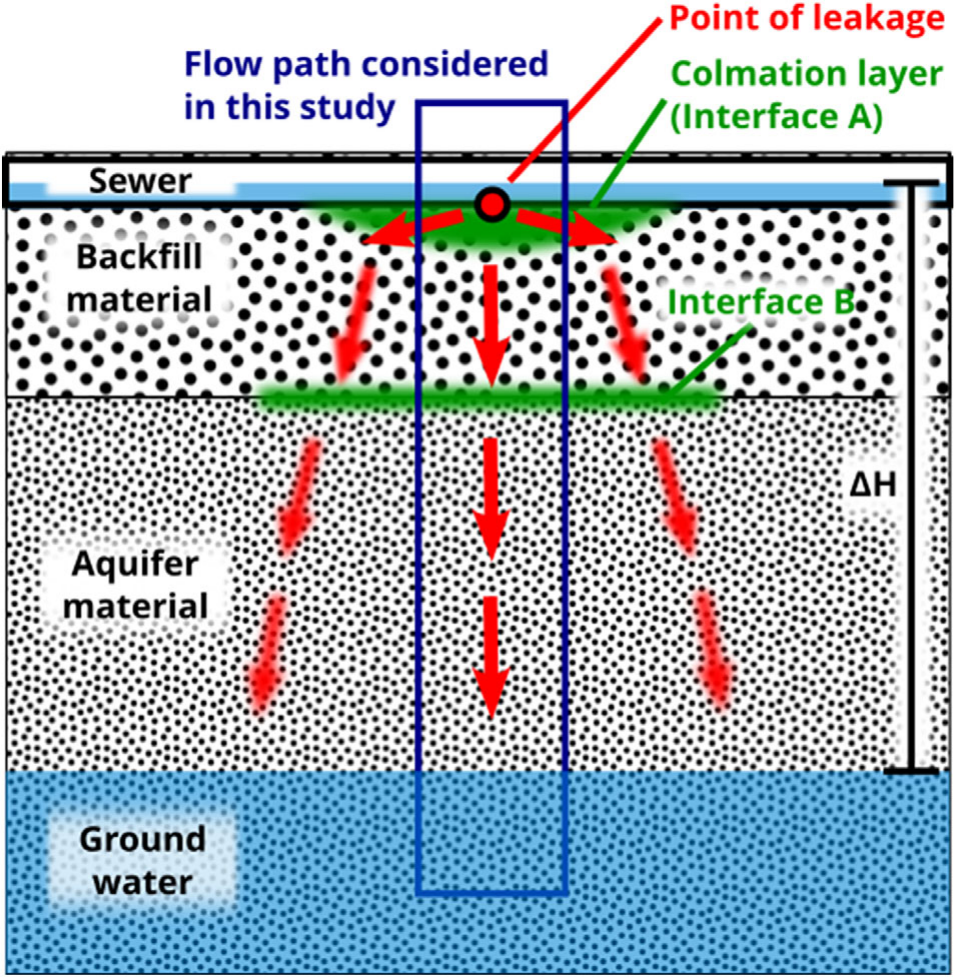
Schematic sketch of conceptual model. Potential lateral flow components due to capillary diffusion or permeability changes at “Interface A” (lower boundary of the colmation layer) or “Interface B” (lower boundary of the backfill material) are omitted.

The present study is restricted to one dimension, even though we acknowledge that sewer leakage is usually happening from a point source into a three‐dimensional, heterogeneous porous medium. However, due to the difficulty in appropriately determining generalized parameters for various inputs connected to sewer leakage—for example, extent of leakage in comparison to natural groundwater recharge or heterogeneity of the different layers—this study is a proof‐of‐concept qualitatively related to real‐world examples. Any lateral flow due to capillary forces or ponding at (sloping) interfaces due to, for example, the capillary barrier effect (Scarfone et al. 2023) between the layers are not considered here. Hence, the determined flow rate and the exact hydraulic disconnection depth in this simulation should always be seen under these restrictions (see Figure 1). Nevertheless, qualitatively, we expect the process of hydraulic disconnection to happen in a similar manner in real‐world 3D scenarios and that its sensitivity to different parameters is equivalent, though absolute numbers might be different.

**Figure 2:**
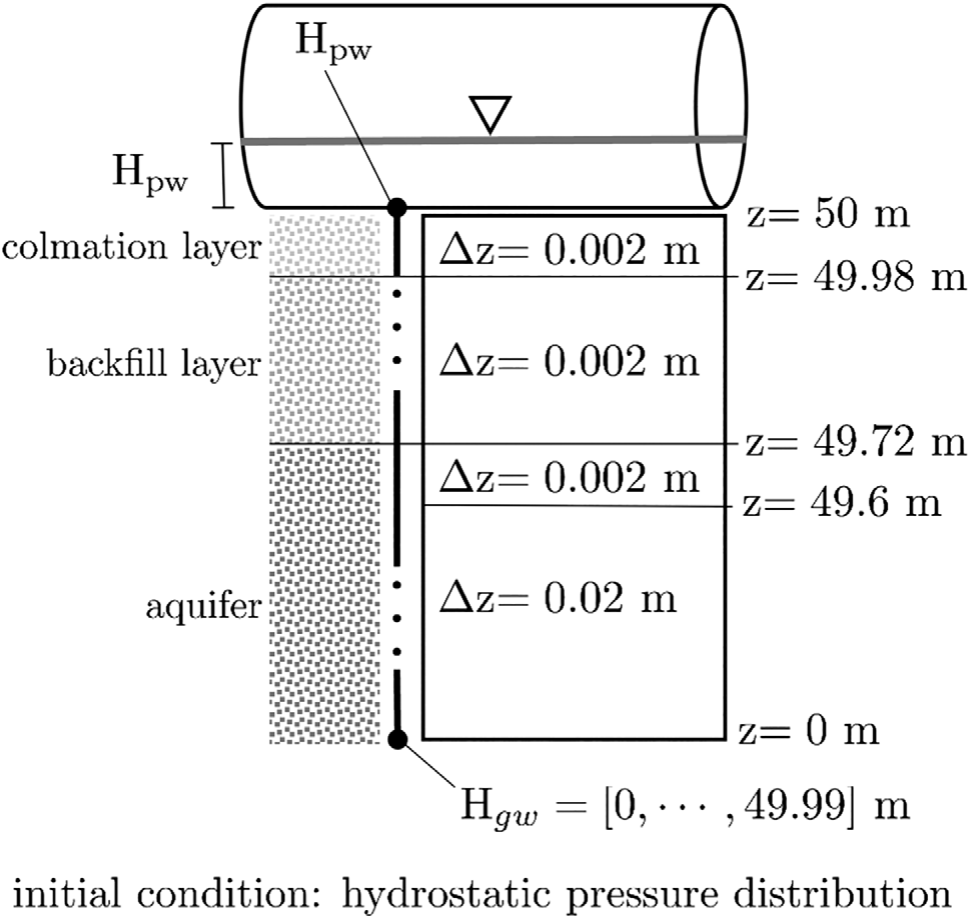
Conceptual model (gw = groundwater; pw = pipe water) with the spatial increment sizes ∆z as determined using the grid convergence study. Note that the hydrostatic head *H*_gw_ is only valid under the assumption of horizontal groundwater flow in the aquifer.

The conceptual model of the base case is visualized in Figure 2. This base case represents pipe leakage into a sandy natural porous medium. In model variations of the base case described later, the natural porous medium was changed to either loamy sand or sandy loam. In the base case and all model variations, hydraulic properties of the colmation layer and backfill material remain the same. A one‐dimensional vertical model domain represents a layered system of in total three materials, namely (and from top to bottom) the colmation layer, the backfill material and the aquifer material. The colmation layer has a thickness of 0.02 m (Ellis et al. 2009; Karpf 2012), the backfill material has a thickness of 0.26 m and the aquifer material has a thickness of 49.72 m. Flow is controlled by two Dirichlet‐type boundary conditions (BC) on the top and bottom nodes of the model domain. A constant hydraulic head of *H*_pw_ = 0.04 m is assigned to the BC on the top node. This value is assumed to be the pipe water level under dry‐weather conditions. The constant head assigned to the bottom node represents the groundwater head above the bottom node. In separate model runs, this value is varied such that it represents groundwater levels of *H*_gw_ = [0.09, …, 49.99] m above the domain bottom. Step size of this groundwater level array is 0.1 m and the uppermost 0.1 m are represented with a finer resolution of 0.05 m. Variation of the bottom BC value leads to different leakage fluxes, which are later analyzed for the hydraulic disconnection of pipe water and groundwater. Initial condition of all simulations is the hydrostatic pressure distribution adjusted for H_gw_. The temporal discretization is increased from small timesteps of ∆*t* = 1 s to ∆*t* = 10,000 s using the automatic proportional‐integral (PI) feedback controller method (Kalbacher et al. 2011) and yielding up to a total simulation time of 1 × 10^10^ s, at which a stationary potential distribution (and leakage flux) has formed. All results shown represent stationary conditions. Note that all values for the leakage flux are taken as absolute values and therefore no longer indicate the direction of the flux. The linear solver used is the Biconjugate Gradient Stabilized method (e.g., Joly and Meurant 1993) with an error tolerance of $1\times 10^{-14}$. The linearization method used is the Picard method (Celia et al. 1990).

**Figure 3:**
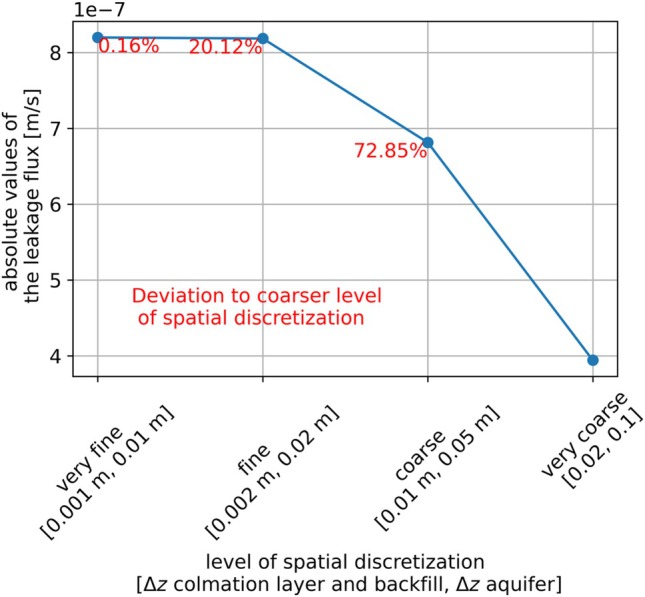
Results of the grid convergence study.

The spatial discretization was determined using a grid convergence study. Individual simulation runs were performed on numerical grids with successively finer spatial discretization. After each model run, the flux across the upper boundary was compared with flux values from the coarser level of spatial discretization. We used the 1% deviation criterion by Graf and Degener (2011) to determine the level of spatial discretization at which the numerical solution becomes independent of the resolution of the spatial discretization. Results and spatial increment sizes are visualized in Figure 3. Clearly, spatial increment sizes of ∆*z* = [0.002, 0.02] m (for the distribution of these increment sizes, see Figure 2 of the conceptual model) are appropriate for the present study.

The base case of the conceptual model was firstly used to analyze both, the hydraulically connected and disconnected phase of the problem. For that purpose, the evolution of the leakage flux over time was analyzed, together with the spatial distributions of the product of relative permeability and saturated hydraulic conductivity, as well as the effective saturation.

Subsequently, in a sensitivity analysis, the base case of the conceptual model was modified, such that it represents the hydraulic disconnection of leaky subsurface pipes under various conditions. For this purpose, values of the pipe water level, the colmation layer thickness, the backfill material thickness, and material properties of all layers (namely, their hydraulic conductivity, effective porosity, and van Genuchten (1980) shape parameters *α* and *m*) were multiplied by 0.5 and 1.5 in order to analyze sensitivity of the hydraulic disconnection depth and leakage flux to those individual variations. Because a multiplication with 1.5 would yield values not coherent with literature data for the van Genuchten (1980) m in the backfill and aquifer material, the initial values of that parameter were multiplied with 1.25.

Lastly, the aquifer material was changed from sand to loamy sand and sandy loam in order to analyze hydraulic disconnection in an aquifer material with different soil properties. All values for pipe water level and material properties of the base case and the different aquifer materials are summarized in Table 1. In these simulations with different aquifer materials, the array of *H*_gw_ was modified (i.e., from of *H*_gw_ = [0.09, …, 49.99] m with a step size of 0.1 m (0.05 m in the uppermost 0.1 m) for a sandy aquifer to *H*_gw_ = [0.09, …, 49.99] with a variable step size and higher step size resolution around the point where hydraulic disconnection occurs for both the loamy sand and sandy loam, respectively) in order to save computation time. The van Genuchten (1980) parameters were partly taken from Carsel and Parrish (1988) and partly determined using the method by Peche et al. (2024) embedded in the computer program HYPAGS (Peche and Houben 2023). Note that the large value for the shape parameter *α* for sand (*α* = 14.5 1/m) from Carsel and Parrish (1988) led to numerical instabilities. Due to inaccessibility of residual saturation data for the backfill material and colmation layer, the residual saturation was globally set to 0.1.

**Table 1:** Fluid Properties and Hydraulic Parameters of the Base Case and the Model Variations with Different Aquifer Material

Name	Parameter	Value	Source
Fluid properties (at *T* = 10 °C)			
Density	*ρ* (kg/m^3^)	999.7	‐
Dynamic viscosity	*μ* (kg m^−1^ s^−1^)	0.001306	‐
Hydraulic parameters			
Aquifer (base case: sand; model variations from the base case: loamy sand; *sandy loam*)			
Hydraulic conductivity	*K*_aquifer_ (m/s)	1.069e‐4; 4.001e‐5; *1.226e‐5*	Carsel and Parrish (1988)
Effective porosity	Φ_aquifer_ (‐)	0.2; 0.15; *0.13*	Han et al. (2008); Woessner and Poeter (2020)
van Genuchten (1980) shape parameters	*α*_aquifer_ (m^−1^)	6.35; 6.21; *3.40*	Carsel and Parrish (1988); Peche et al. (2024)
	*m*_aquifer_ (‐)	0.65; 0.31; *0.41*	Carsel and Parrish (1988); Peche et al. (2024)
Backfill material			
Hydraulic conductivity	*K*_backfill_ (m/s)	7.836e‐4	Carsel and Parrish (1988); Karpf (2012)
Effective porosity	Φ_backfill_ (‐)	0.14	Karpf (2012)
van Genuchten (1980) shape parameters	*α*_backfill_ (m^−1^)	6.35	Peche et al. (2024)
	*m*_backfill_ (‐)	0.627	Carsel and Parrish (1988)
Colmation layer			
Hydraulic conductivity	*K*_colmation layer_ (m/s)	1.306e‐7	Karpf (2012)
Effective porosity	Φ_colmation layer_ (‐)	0.02	Karpf (2012)
van Genuchten (1980) shape parameters	*α*_colmation layer_ (m^−1^)	2	Carsel and Parrish (1988); Karpf (2012)
	*m*_colmation layer_ (‐)	0.29	Carsel and Parrish (1988); Karpf (2012)

### Increase of Result Resolution by Interpolation

**Figure 4:**
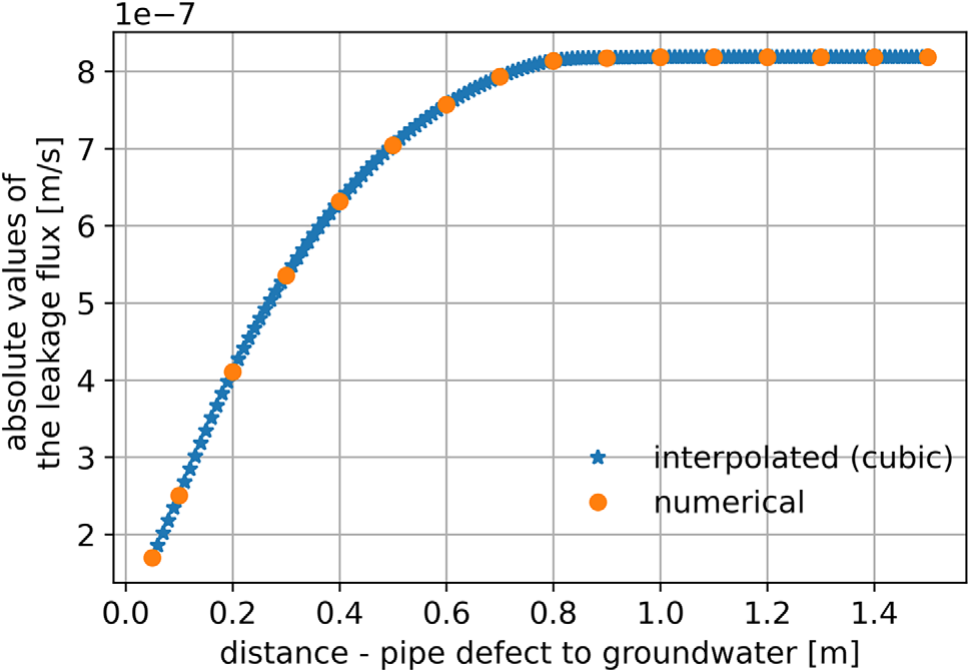
Base case: leakage flux over distance of pipe defect to groundwater. Orange: results of the numerical model; blue: results of the cubic interpolation.

In order to analyze the depth from pipe defect to groundwater at which hydraulic disconnection occurs and the respective leakage flux with a higher resolution than the 0.05 m and 0.1 m resolution represented by the bottom boundary condition array, the result resolution was refined by cubic interpolation between results from the numerical model. Resolution of the interpolated values is 0.01 m. This method decreased the total simulation time of all model variations by approximately a factor of 10. The resolution of both, numerical model results and interpolated values is visualized in Figure 4.

## Results

### Base Case

Results of the base case in form of absolute leakage flux over distance from pipe defect to groundwater level are visualized in Figure 4. Note that values of the leakage flux are oriented in negative *z*‐direction and therefore values are negative. However, we use absolute values throughout the present document for the sake of simplicity. With decreasing groundwater level, leakage flux increases. This increase is nonlinear until, at a groundwater level depth of 0.89 m the leakage flux converges to a quasi‐constant value of 8.17·10^−7^ m/s. Lowering the groundwater level further only incrementally increases that value by <0.1%, which is why we define that state as hydraulically disconnected.

**Figure 5:**
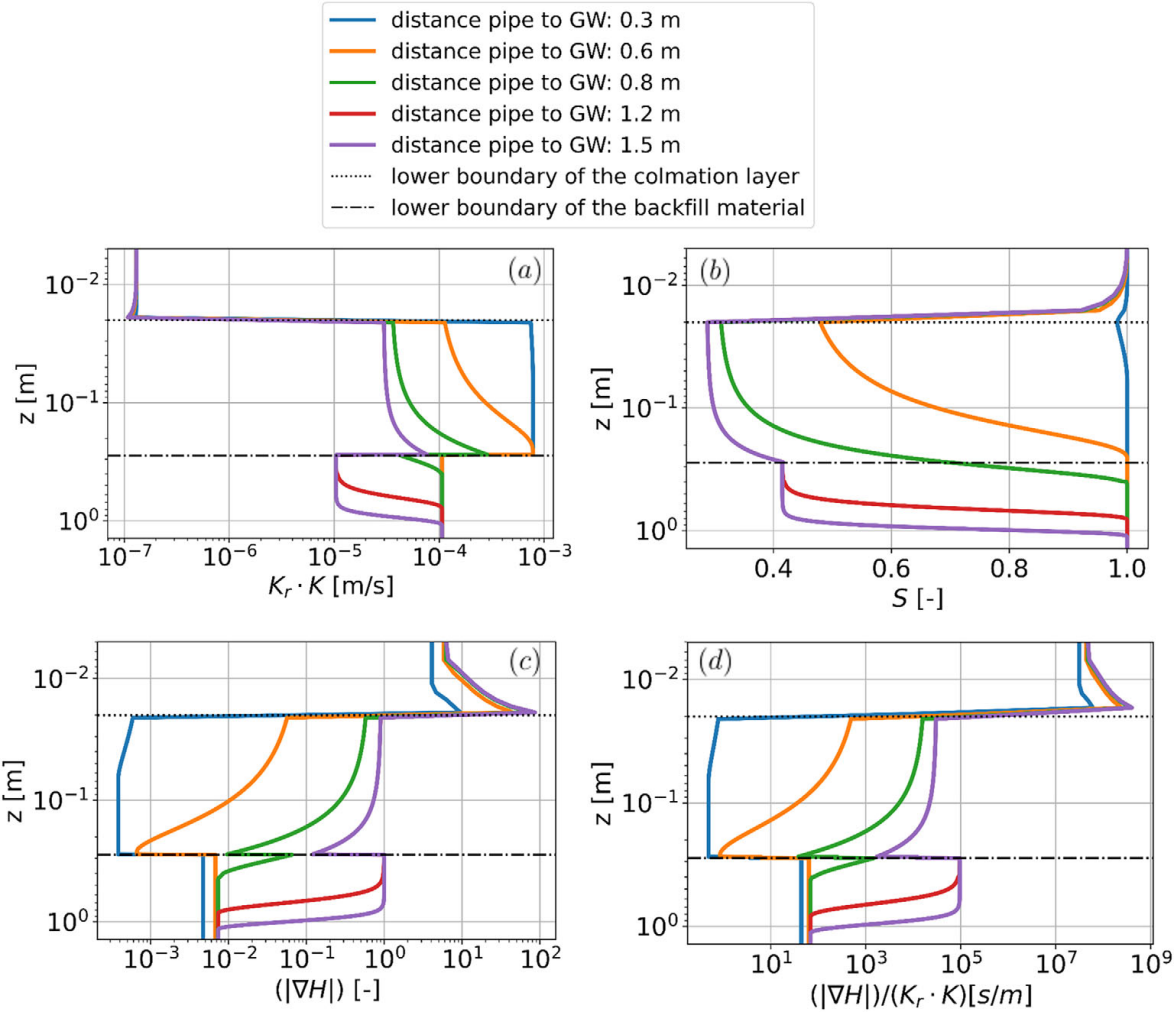
Base case: Distributions of (a) the product of relative and hydraulic conductivity, (b) effective saturation, (c) hydraulic gradient, and (d) ratio of hydraulic gradient and relative conductivity over depth (log‐scaled). Note that the boundaries at which a parameter contrast (between colmation layer and backfill material and backfill material and aquifer) is visualized in dashed lines. Different colors indicate the state of hydraulic connection, imposed by different distances from pipe defect to groundwater level, where red and purple colors represent conditions at hydraulic disconnection.

In Figure 5, the spatial distributions of the product of relative and hydraulic conductivity $K_{r}K$ (Figure 5a), the effective saturation S (Figure 5b), absolute values of the grid cell‐wise calculated hydraulic gradient ∣∇H∣ (Figure 5c) are visualized as a function of depth. In order to analyze the dominating impact of either, ∣∇H∣ or $K_{r}K$, the ratio of both is also shown (Figure 5d).

The physical interpretation of the present three‐layered problem as visualized in Figure 5 is complex. In order to maintain a constant flux, this interplay of relative permeability and hydraulic gradient between all porous media changes with a different conceptual model (e.g., boundary conditions, thicknesses of the respective layers, material properties).

In our case, $K_{r}K$ and S over depth show similar distributions and with decreasing groundwater levels, values for both decrease as they are positively correlated through the van Genuchten (1980) functions.

At a hydraulically disconnected state (distance pipe to GW: 1.5 m), about half (the topmost 0.008 m) of the colmation layer is fully saturated with $K_{r}K=K,\;K_{r}=1$. However, the remaining part of the colmation layer is characterized by a decreasing S and thus $K_{r}<1$. Directly above the interface from colmation layer to backfill material, the effective saturation has reduced to S=0.29, yielding a $K_{r}K\approx 1.25\cdot 10^{-7}$ m/s.

Directly below the interface, the backfill material approaches a unit gradient and is close to a state of hydraulic disconnection, with $K_{r}K$ increasing to $2.98\cdot 10^{-5}$ m/s while S remains at 0.29.

At the model boundary between the pipe and the colmation layer the influx from the pipe is determined by the saturated conductivity K in the colmation layer and the near constant (with depth) gradient ∣∇H∣ below this boundary (Figure 5a and 5c). Due to the Dirichlet boundary condition, a saturated flow develops in the upper colmation layer, as influx is not limited by water shortage. In the backfill material layer below, the flux must be maintained for reasons of mass conservation. To achieve this, the saturation in this layer just below this interface must decrease to the point on the conductivity/saturation curve of the backfill material at which $K_{r}K$ ensures this flux at the given |∇H|. The latter is determined here by the constraint of approaching hydraulic disconnection (free drainage/unit gradient). Due to the limited thickness of the backfill material layer and the resulting proximity to the next interface, desaturation to unit gradient is prevented below this interface, but |∇H| is very close.

Only at this saturation can the flux be maintained under the given conditions. Technically, this requirement is met by draining the colmation layer at the interface to the required saturation. As long as the saturation is higher, the backfill material would conduct more water downwards than is replenished by the flux from the colmation layer. To also maintain the constant flux in the resulting unsaturated lower part of the colmation layer, |∇H| must increase to its maximum value of |∇H|=86.50 as can be seen in Figure 5c.

Further downwards in the backfill material, conductivity and effective saturation increase up to the next interface between backfill material and aquifer (Figure 5a and 5b). As can be seen in Figure 5c, below this interface, the aquifer is in a state of hydraulic disconnection (free drainage/unit gradient). The limiting values for this increase are $K_{r}K=1.05\cdot 10^{-5}$ m/s and S=0.42 which describe the point on the conductivity/saturation curve of the aquifer material at which $K_{r}K$ equals flux as |∇H| is equal to one (unit gradient). To compensate for the increasing saturation in the backfill material (and the resulting increase in $K_{r}K$) up to this interface, |∇H| must decrease as can be seen in Figure 5c.

Finally, in the aquifer material, $K_{r}K$ and S increase up to their maximum values while approaching the groundwater level. At the same time |∇H| decreases from unit gradient to 0.0074 to maintain the flux. Qualitatively, relative conductivity distributions are similar to a previous study by D'Aniello et al. (2021).

In the hydraulically connected stage, the hydraulic gradients are the lowest in all layers, as the capillary fringe of the high groundwater level leads to the consistently highest saturations and conductivities throughout the profile. The combination of high conductivities and low gradients throughout the profile reduces the influx from the pipe at the upper boundary—a behavior that is expected for the hydraulically connected stage. Generally, for the hydraulic disconnected cases with a distance of pipe to groundwater ≥0.89 m (valid for both cases distance pipe to GW = [1.2, 1.5] m in Figure 5), distributions of |∇H|, $\left(K_{r}K\right)$ and S over depth overlap for most parts of the profile. The closer the curves come to fulfill the condition of a hydraulically disconnected stage, the more they nestle against each other.

### Sensitivity Analysis

**Figure 6:**
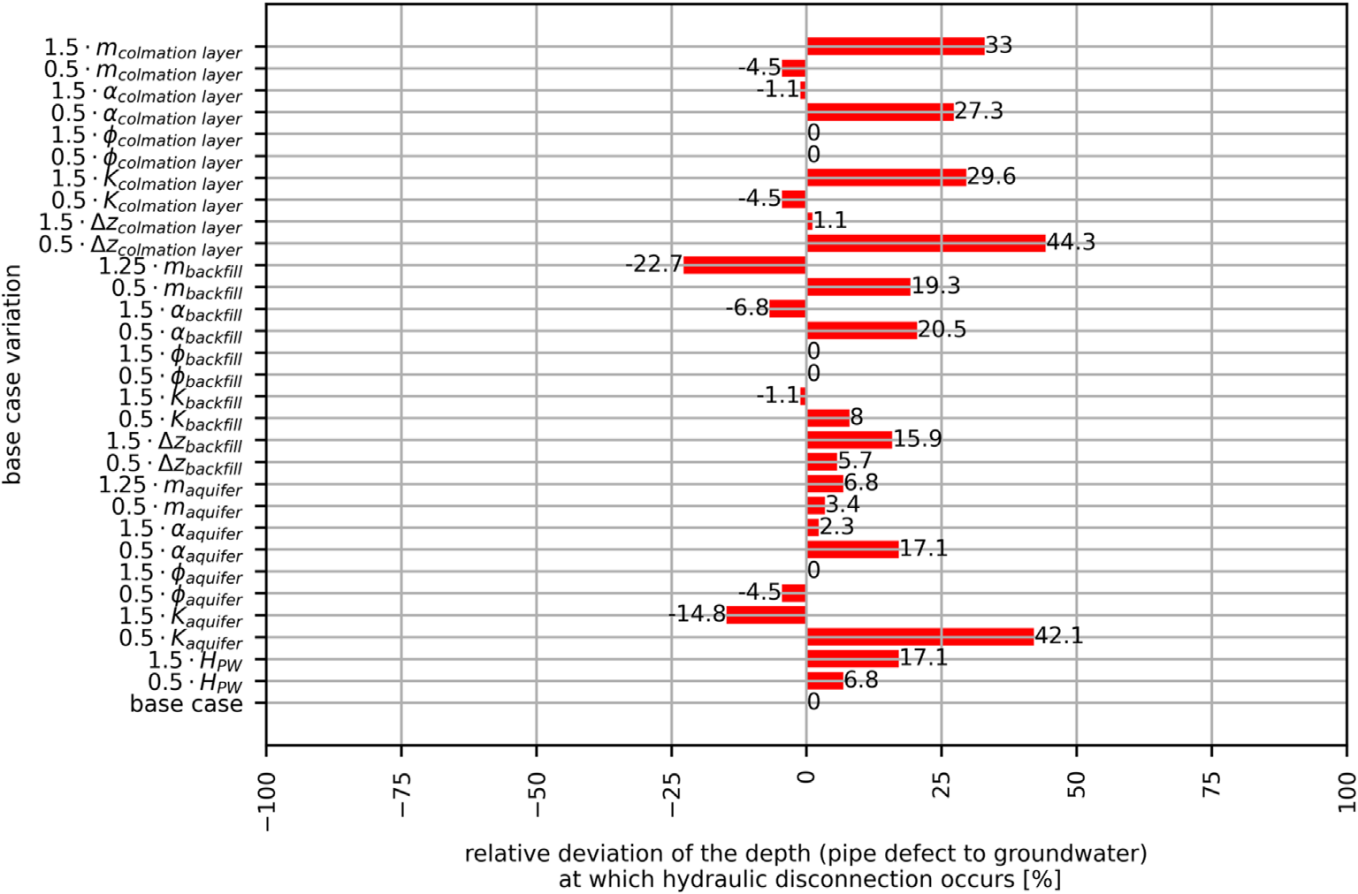
Sensitivity analysis regarding the depth at which hydraulic disconnection occurs.

**Figure 7:**
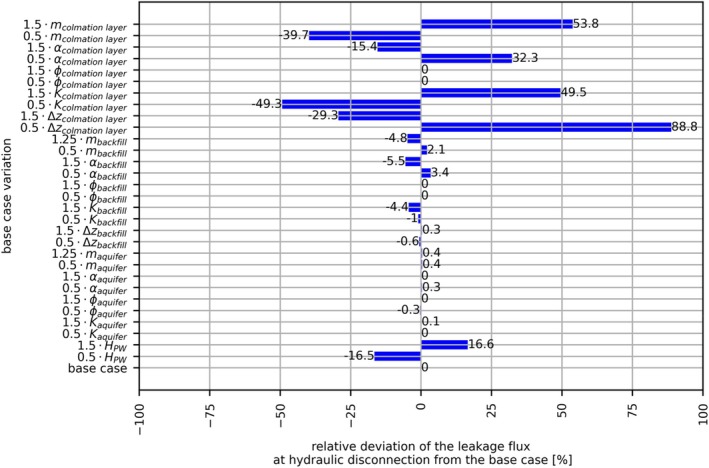
Sensitivity analysis regarding the leakage flux at hydraulic disconnection.

**Figure A2:**
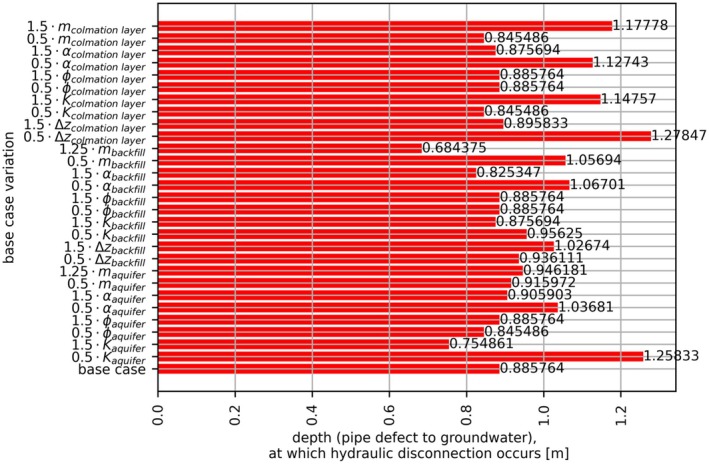
Sensitivity analysis regarding the depth at which hydraulic disconnection occurs—absolute values.

**Figure A3:**
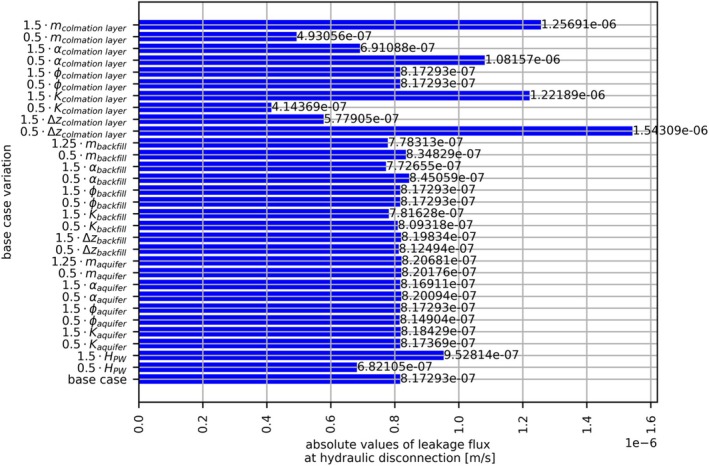
Sensitivity analysis regarding the leakage flux at hydraulic disconnection—absolute values.

Results of the sensitivity analysis in form of the relative impact on the hydraulic disconnection depth (depth from pipe defect to the groundwater level at which hydraulic disconnection occurs) and leakage flux are visualized in Figures 6 and 7, respectively. Results in form of absolute values are visualized in the Appendix (Figures A2 and A3).

For the hydraulic disconnection depth, as visualized in Figure 6, the most sensitive parameters leading to a >40% increase are a decrease in the thickness of the colmation layer, a decrease in the hydraulic conductivity of the aquifer. Increasing the van Genuchten (1980) m and decreasing the *α* of the colmation layer, respectively, as well as increasing the hydraulic conductivity of the colmation layer leads to an increase of the hydraulic disconnection depth by >25%. Further sensitive parameters are the van Genuchten (1980) m and *α* of the backfill material, the thickness of the backfill material, the van Genuchten (1980) *α* of the aquifer material and the pipe water level. A variation of remaining parameters leads to a change in hydraulic disconnection depth of less than 7%.

For the leakage flux, as visualized in Figure 7, the pipe water level and all hydraulic parameters of the colmation layer except the effective porosity are sensitive. Hydraulic parameters of the backfill material and aquifer material lead to a maximum result deviation of 5.5% and 0.6%, respectively.

It is interesting that the sensitivity analyzes of both, depth at which hydraulic disconnection occurs and leakage flux do not correlate. This leads to the conclusion that a parameter variation may lead to a change in the disconnection depth while the leakage flux stays constant and vice versa.

### Hydraulic Disconnection in Different Aquifer Porous Media

**Figure 8:**
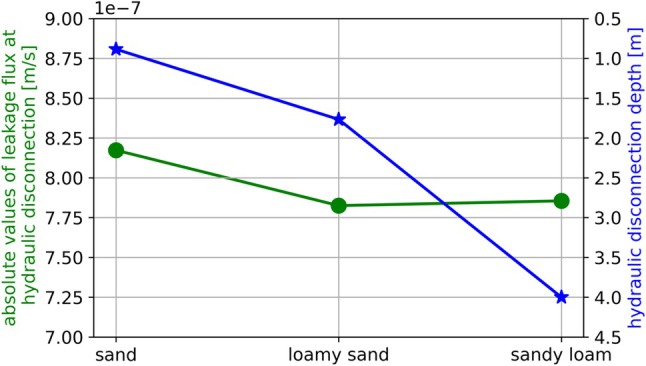
Hydraulic disconnection of leaky pipes and groundwater for different soil types and in form of leakage flux at hydraulic disconnection (green) and depth (distance of pipe defect to groundwater) at which hydraulic disconnection occurs (blue).

**Figure A4:**
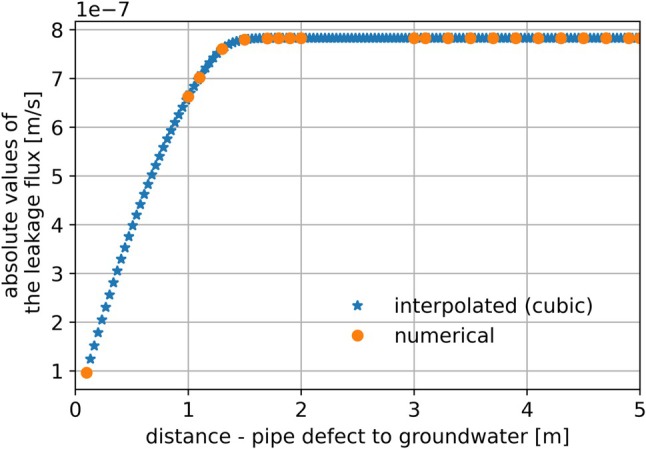
Model variation—loamy sand aquifer: Results in form of leakage flux over distance of pipe defect to groundwater.

**Figure A5:**
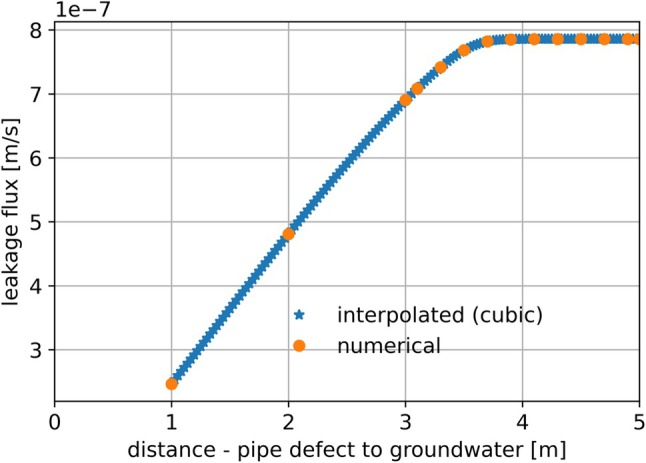
Model variation—sandy loam aquifer: Results in form of leakage flux over distance of pipe defect to groundwater.

Additional to the base case, further simulations were carried out in a loamy sand and in a sandy loam material. Colmation layer and backfill material properties are constant in all model variations and the base case. Results in form of leakage flux and depth from pipe defect to groundwater level at which hydraulic disconnection occurs for the three aquifer materials are visualized in Figure 8. Further results for the two model variations in form of leakage flux over depth from pipe defect to groundwater level are visualized in Figures A4 and A5 in the Appendix. Qualitatively, one can conclude that the aquifer material greatly influences the characteristics of hydraulic disconnection. A less permeable aquifer material leads to an increase of the depth at which hydraulic disconnection occurs. For a sandy aquifer, that depth is at ~0.89 m, while for loamy sand and sandy loam, that value increases to ~1.77 m and 4.00 m, respectively. This is due to a higher water retention capacity of finer textured porous media against gravity leading to a larger disconnection depth.

For the absolute leakage flux, the results are less intuitive. For a sandy aquifer, leakage flux at the state of hydraulic disconnection equates to 8.18·10^−7^ m/s. For loamy sand and sandy loam, this value equates 7.83·10^−7^ and 7.86·10^−7^ m/s (compare with similar values for the effective saturation of 0.4, 0.83, and 0.82), respectively. The latter flux is slightly larger for the finer textured sandy loam, as at unit gradient the corresponding water content is slightly lower, increasing the stationary pipe drainage due to a slightly stronger desaturation spreading upwards and the resulting slight increase in gradient in the unsaturated part of the colmation layer to maintain flow. The previously described sensitivity analysis indicates that the van Genuchten (1980) parameters have only little influence on leakage flux. Generally, the difference in leakage flux for both model variations is very small (<0.4%). The relative deviation to the base case model with a sandy aquifer material is <5%.

## Discussion

The present novel proof‐of‐concept study shows that leaky pipes can hydraulically disconnect from underlying groundwater. The concept can be analogously seen to the hydraulic disconnection of rivers from groundwater, except that it is a three‐layered problem consisting of colmation layer, backfill material and aquifer material. The hydraulic disconnection of leaky sewers has implications for numerical modelers. A certain depth from the leaky pipe to the groundwater level leads to a hydraulically disconnected stage. This implies a constant leakage flux, even when the groundwater level is lowered further. As a consequence, the leakage flux becomes independent from groundwater height, which means that the calculation of subsurface flow can be neglected as long as the material properties of the three‐layered problem remain the same. In such a stage, leakage fluxes are only dependent on the pipe water level. Hence, for numerical modeling of such a problem under hydraulically disconnected conditions, only the computation of pipe flow is necessary. However, extensive testing is a prerequisite for dismissing subsurface flow calculations, particularly given that fluctuating groundwater levels can re‐establish hydraulic connectivity in previously disconnected systems. It should be noted that the calculations in this study are derived from a specific conceptual model incorporating simplifications of real‐world complexities. Consequently, our findings should be interpreted primarily in a qualitative context.

The three‐layered problem used in the present study may only apply to certain locations. For example, D'Aniello et al. (2021) conceptualized the pipe leakage problem as a different three‐layer system consisting of the backfill material, embedment material and the ambient aquifer material. The impact of an additional embedment possibly introduces another contrast in hydraulic parameters and thus leads to different results. However, we assume our three‐layer system to represent typical conditions in Germany and many other countries.

For the current pipe leakage problem, we demonstrate and analyze the complex interplay between the hydraulic gradient and the product of relative permeability and hydraulic conductitivity at a constant flux. Our study shows that, at a given flux, the distributions of the hydraulic gradient and effective saturation do not show the behavior of a capillary barrier (Scarfone et al. 2023; Chetti et al. 2024) with an increase of saturation directly above the interfaces between fine and coarse porous media. The latter phenomenon occurs when the constant flux over an interface can only be maintained if an excess pressure—by the formation of a saturated zone above the interface—builds up. It can be assumed that a capillary barrier may form if the conductivity contrast between backfill and aquifer material exceeds the difference between backfill material and colmation layer.

It is also interesting that a unit gradient (free drainage or gravity drainage; Sisson and van Genuchten 1991) develops in the aquifer material under hydraulically disconnected conditions. For the current problem, the hydraulic gradient approaches but never reaches unit gradient in the backfill material. It is assumed that a unit gradient will form in the backfill material if its thickness increases.

Our study shows that an unsaturated zone forms within the colmation layer with a thickness of 2 cm. This questions the validity of a zero dimensional model representing the colmation layer and utilizing Darcy's law when modeling the exfiltration flux (e.g., Ellis et al. 2009; Karpf and Krebs 2011). The unsaturated conditions may be adequately represented by the proportionality factor of these equations, if calibrated/chosen correctly, but varying thicknesses of the unsaturated zone may not be captured. Thus, to capture the subsequent nonlinearity of fluxes, the best approach would be a nonlinear model representing the thickness and effective saturation of the unsaturated part of the colmation layer, possibly calculated with the Richards (1931) equation or a nonlinear leakage function (Peche et al. 2017). The latter, if different properties than the ones in Peche et al. (2017) are considered, however requires extensive numerical calculations. Furthermore, the most sensitive parameter for the depth from pipe defect to the groundwater level at which hydraulic disconnection occurs is the thickness of the colmation layer. While a decrease in thickness (relative to the 2 cm thickness assigned for the model base case) has a large effect on the disconnection depth (plus ~44%), an increase leads to an increase in disconnection depth by only ~1%. This is because a lower thickness leads to a considerable increase in flow, which counteracts drainage up to the unit gradient. The colmation layer thickness in literature is described to be up to 5 cm (Ellis and Bertrand‐Krajewski 2010) and is often assumed to be at least as thick as the pipe wall.

The sensitivity analysis shows the importance of choosing the hydraulic parameters judiciously. While the parameters of backfill material are well known and parameters of the aquifer material can be determined, especially the parameters of the colmation layer are often unknown and generally difficult to determine due to inaccessibility. Results from different studies (Okubo and Matsumoto 1983; Rauch and Stegner 1994; Blackwood et al. 2005; Dohmann 2013) indicate that the colmation layer properties are highly variable (at times by orders of magnitude) and depend on site‐specific conditions (e.g., physico‐chemical wastewater signature, soil type, pipe water level). This highlights the challenge of choosing adequate hydraulic parameters. Also, the assumptions that these parameters are independent of time may not be true because colmation layers may break up at large hydrostatic pressures in the pipe and parameters may vary in time due to temporally changing physico‐chemical wastewater signatures leading to varying biofilm growth in the pore space (which reduces pore channel cross‐sectional area and directly impacts flow). Cyclic parameter variations attributed to biofilm life cycles may also occur.

Varying aquifer materials by calculating leakage into a sand‐, loamy sand‐ and sandy loam aquifer shows that the aquifer material significantly impacts the depth between pipe defect and groundwater level at which hydraulic disconnection occurs. Generally, one may state that a finer textured aquifer material leads to a lower groundwater level necessary to hydraulically disconnect the leaky pipe from groundwater. This does not apply for the leakage flux. In fact, while the leakage flux for the base case (sandy aquifer) was significantly larger than the other two model variations (loamy sand aquifer, sandy loam aquifer), there was only a small deviation in leakage flux between the loamy sand‐ and sandy loam aquifer model variations. This small deviation is likely due to the different hydraulic properties.

The present study is based on major assumptions and simplifications. Primarily, all calculations are done in one spatial dimension. While in reality, a radial flow field forms around a leaky pipe and water spreads laterally, our one‐dimensional approach merely allows water movement into that one dimension. This leads to an overestimation of the saturation in that dimension and thus an overestimation of exfiltration rates. Further, and as discussed above, a major simplification is that colmation layer properties are assumed to be constant. In reality, colmation is highly dynamic (e.g., Vollertsen and Hvitved‐Jacobsen 2003; see above for more references) and our approach ignores growth, breaking‐up, aging and self‐sealing effects, each affecting its hydraulic properties. Another simplification of our approach is that porous media are assumed to be homogeneous and isotropic. Our approach represents a spatially relatively large‐scale mean of small‐scale heterogeneities (Warren and Price 1961) on the spatial scale of the continuum. By using a one‐dimensional approach, we neglect the tensor behavior of hydraulic properties. This leads to an idealized flow field in which the specific flux vector is directed into the same direction as the potential gradient. Another major simplification is the constant pipe water level. In a residential area, that value varies cyclically on a diurnal basis with human activity, also weekday‐weekend variations (Enfinger and Stevens 2006). In combined sewer systems, precipitation and runoff events further impact sewer water levels and can exceed the capacity of the pipe network in extreme cases (Peters and Zitomer 2021). However, Peche et al. (2017) show that a timescale discrepancy between pipe flow and variably saturated flow in the porous medium exists. This may justify the use of an averaged pipe water level because the response time in the porous medium is usually much slower than the short pipe flow events. Clearly, the model used in the presents study is a broad abstraction of a real system (Anderson et al. 2002). However, despite all simplifications and limitations listed above, we believe it is qualitatively accurate for the proof of concept of the hydraulic disconnection of leaky sewers from groundwater.

## Conclusions and Outlook

Conclusions of the present study can be summarized as follows:

- Leaky pipes can hydraulically disconnect from groundwater if the groundwater level is low enough. If the groundwater level is further lowered, the leakage flux stays constant as long as the material properties stay the same.
- Numerical models of the pipe leakage problem under hydraulically disconnected conditions can be reduced to the pipe flow domain since, provided the material properties remain constant, the leakage flux depends only on the pipe's water level. The subsurface flow domain may then be neglected, which leads to a highly increased numerical efficiency.
- Under dry‐weather flow conditions, assuming no significant changes in the physicochemical signature of the sewage and hydraulic properties, the leakage flux and associated contaminant mass flux under disconnected conditions stays constant. This limits the soil and groundwater contamination.
- It is crucial to judiciously choose adequate hydraulic parameters, because the parameters of the colmation layer are highly sensitive.
- Under unsaturated conditions the general assumption of full saturation in the colmation layer is questionable and applies only under certain conditions (e.g., rather small contrast to the backfill material properties, possibly large hydrostatic pressures in the pipe).
- A less permeable aquifer material with different van Genuchten (1980) parameters leads to an increase of the depth from pipe defect to groundwater level at which hydraulic disconnection occurs.

In future studies, the methodology used in the present study may be extended to three spatial dimensions in order to quantitatively analyze the hydraulic disconnection of leaky sewers from groundwater. Also, it would be interesting to investigate the hydraulic disconnection in case studies representing a real system and over a longer period of time (possibly annual) in order to detect reoccuring (possibly seasonal) patterns. While this study provides a conceptual framework, the precise quantification of hydraulic disconnection under complex field conditions remains a subject for future research.

## Authors' Note

The authors do not have any conflicts of interest or financial disclosures to report.

## Data Availability

All data, models, and code that support the findings of the present study are available from the corresponding author upon request.

## Mathematical Model

In the present study, Richards (1931) equation is used in its one‐dimensional and one‐primary variable form. The primary variable is water pressure *p* (M L^−1^ T^−2^). That equation reads:

$$\phi \rho \frac{\partial S}{\partial p}\frac{\partial p}{\partial t}-\frac{\partial }{\partial z}\cdot \left[\rho \frac{K_{r}K}{\mu }\left(\frac{\partial p}{\partial z}-\rho g\right)\right]=\frac{\rho \cdot Q}{V}$$

where ϕ (‐) is the effective porosity, ρ (M L^−3^) is fluid density, t (T) is time, z (L) is the vertical spatial coordinate, μ (M L^−1^ T^−1^) is fluid dynamic viscosity, g (L T^−2^) is gravitational acceleration, Q (L^3^ T^−1^) is a source term and V (L^3^) is unit volume.

The soil water pressure as a function of effective saturation after van Genuchten (1980) is calculated using

$$-p=\frac{\rho g}{\alpha }{\left[S^{\frac{-1}{m}}-1\right]}^{\frac{1}{n}}$$

where α (L^−1^) is a shape parameter and further shape parameters *m* (‐) and *n* (‐) are related by $m=1-\left(n^{-1}\right)$. The relative permeability is calculated using

$$K_{r}=S^{\frac{1}{2}}{\left[1-{\left(1-S^{\frac{1}{m}}\right)}^{m}\right]}^{2}$$
